# Antimicrobial multidrug resistance in the era of COVID-19: a forgotten plight?

**DOI:** 10.1186/s13756-021-00893-z

**Published:** 2021-01-29

**Authors:** Eric Pelfrene, Radu Botgros, Marco Cavaleri

**Affiliations:** 1 Office of Vaccines and Therapies for Infectious Diseases, Human Medicines Division, European Medicines Agency, 1083 HS Amsterdam, The Netherlands; 2 Biological Health Threats and Vaccines Strategy, Clinical Studies and Manufacturing Task Force, European Medicines Agency, 1083 HS Amsterdam, The Netherlands

**Keywords:** Antibiotics, Antimicrobial resistance, Antimicrobial stewardship, Coronaviruses, COVID-19, One health, Pandemic, SARS-CoV-2

## Abstract

**Background:**

Antimicrobial resistance (AMR) is a growing global problem to which the ongoing COVID-19 pandemic may further contribute. With resources deployed away from antimicrobial stewardship, evidence of substantial pre-emptive antibiotic use in COVID-19 patients and indirectly, with deteriorating economic conditions fuelling poverty potentially impacting on levels of resistance, AMR threat remains significant.

**Main body:**

In this paper, main AMR countermeasures are revisited and priorities to tackle the issue are re-iterated. The need for collaboration is stressed, acknowledging the relationship between human health, animal health and environment (“One Health” approach). Among the stated priorities, the initiative by the European Medicines Regulatory Network to further strengthen the measures in combatting AMR is highlighted. Likewise, it is asserted that other emerging health threats require global collaboration with the One Health approach offering a valuable blueprint for action.

**Conclusion:**

The authors stress the importance of an integrated preparedness strategy to tackle this public health peril.

## Background

The viral pandemic caused by Severe Acute Respiratory Syndrome Coronavirus 2 (SARS-CoV-2) has put an enormous strain to the health and economies globally. One of the consequences of these woes was that some essential, lifesaving medicines became short in supply, mainly as a result of temporary lockdowns of manufacturing sites, export bans and stockpiling by local and national authorities. Simultaneously, the pandemic caused an increased in-hospital demand for analgesics, muscle relaxants, anaesthetics and indeed antimicrobial agents [1]. Whilst general unavailability of life-saving products may have led to needless suffering in some of the poorest societies, other regions registered an intense usage of these products in inpatients, including antibiotics [2].

The high number of patients suffering from Coronavirus disease 2019 (COVID-19) worldwide admitted to hospital wards and intensive care units (ICU) are indeed most vulnerable for secondary infections. Against a background risk of circulating bacterial strains and of having invasive procedures as putative portals of infection, worsening inflammatory parameters might make it challenging for the clinician to discern COVID-19 severe inflammatory reaction from bacterial secondary infection, setting a low bar in commencing empirical broad-spectrum antibiotic coverage [3]. Witness to the substantial antibiotic therapy use in hospitalised COVID-19 patients, Rawson et al*.* summarized findings from a series of mainly Chinese reports comprising the period of January to mid- April 2020, revealing that 72% (1450 out of 2010) of patients received broad-spectrum antimicrobials [4]. Although antibiotic prescribing practices might differ between geographic areas, increased work pressure and mental strain on healthcare professionals render it more difficult in adhering to local hospital antimicrobial stewardship policy [5]. Notwithstanding the fact that empiric antibiotics administration negatively influences subsequent diagnostic yield, accumulated evidence though points towards a relatively small proportion of COVID-19 patients suffering from bacterial co-infections. Based on a systematic review involving 2834 patients, Lansbury et al. report co-infection in 7% of hospitalised and 14% of patients admitted to mixed ward/ ICU setting [6]. These figures correspond well to findings by Langford et al*.,* obtained from a living systematic review on bacterial co-infection (at presentation) and secondary in-hospital acquired infection in patients with COVID-19 [7]. Hitherto, the findings on community-acquired bacterial co-infection favourably compare to the figures obtained during seasonal and pandemic influenza episodes, for which nasopharyngeal colonising pathogens such as *S. pneumoniae, S. pyogenes* and *S. aureus* typically cause secondary infections and represent the reason for hospital admission [8–10]. In contrast, for COVID-19, hospital- and ventilator- acquired pneumonia (HAP/VAP) seem prominent, with isolates likely to reflect the local institutional ecology and antimicrobial resistance pattern [7, 11]. Some reports also noted a propensity for putative invasive pulmonary aspergillosis in the most critically ill, prompting physicians to maintain a high level of clinical suspicion [12, 13]. Further prospective monitoring of coinfections / superinfections will be required to provide a better understanding of such risk, and to enable enhanced antimicrobial stewardship [11, 14]. Protocols for prevention, early detection and treatment of infectious complications need to be adhered to, and principally, antimicrobial stewardship programmes need to be integrated into future emergency response preparedness efforts [15]. Ultimately, the intense hospital use of antibiotics in COVID-19 patients at the early stage of the pandemic may influence the level of bacterial resistance [16], impacting the burden of disease well into the future.

Separately, the coronavirus pandemic has triggered a severe and widespread global economic meltdown and risks fuelling a dramatic rise in poverty levels around the globe [17]. Populations residing in lower- and middle-income countries (LMICs) are expected to suffer disproportionally, with many in a vulnerable situation being pushed into penury. Crowded living conditions with poor sanitation, insufficient access to quality healthcare and often irrational use of easily available over the counter (OTC) antimicrobial agents,—possibly of substandard quality—, contribute to gut colonisation and spread of resistant microorganisms [18, 19]. These factors add to the menace of AMR and make the need for the “call to arms” to be timely re-iterated more acute.

## AMR and countermeasures

### Antimicrobial stewardship and educational measures

Following the O’Neill review and recommendations in 2016 to counter the AMR crisis [20], important progress has been made. International initiatives such as the annual World Antibiotic Awareness Week raise the understanding about AMR and the need for prudent use of antibiotics [21]. Emphasis placed on hygiene measures with more frequent handwashing (e.g. through Hand Hygiene Day) seems to pay off. With renewed attention during the current pandemic in relation to correct handwashing techniques, it can be hoped that this will translate into long lasting behavioural change.

As part of the broader agenda, medical professionals should nevertheless continue to avoid inappropriate prescription; a message heeded with mixed success over the last few years [22], and which merits to be re-emphasized during the current pandemic. Huttner et al*.* recently called on the medical community not to neglect antimicrobial stewardship, and to minimize the unintended consequences of inadequate antimicrobial use: the emergence of resistance and added unnecessary toxicity. Antibiotics need to be used responsibly and sparingly, administering empirical therapy to those cases having a high likelihood of bacterial infection, considering local resistance data. Empirical prescription needs to be rapidly re-evaluated and adapted according to microbiological results [23].

### One Health policy

Reducing the use of antibiotics in food-producing animals seeks to preserve the current and future benefit of antibiotics for humans [24, 25]. In this respect, the European Union (EU) prohibited the use of antibiotics as growth promotor in animal feed in 2006 [26]. Since then, significant further progress has been made. The latest European Surveillance of Veterinary Antimicrobial Consumption (ESVAC) report published in October 2020 indicates that sales of antibiotics for use in animals in the European Economic Area and Switzerland fell by more than 34% in the period between 2011 and 2018 [27]. With carbapenem-resistant *Enterobacteriaceae* (CREB) resistant to almost all antibiotics [28], focus has been placed on last resort antibiotics in human infection, such as polymyxins (chiefly colistin). The gene, *mcr-1,* has been found in human and animal isolates of *Enterobacteriaceae* and represents the first known plasmid-mediated resistance to polymyxins [29]. As such, rapid interspecies spread of colistin resistance would be a disastrous evolution. Considering this threat, adding colistin to animal feed has been heavily restricted or discontinued altogether [30–32]. Progress on tackling AMR is however uneven and in animals as food sources, resistance levels are reported to increase in various LMICs hotspots [33].

With the threat of resistance acquisition from animal species and with wildlife identified as a reservoir, continuing to follow the integrated multisectoral One Health approach,—viewed as an issue impacting humans, livestock, aquaculture, wildlife and the environment—, has become imperative [34]. This is also underlined in the recently published EU Pharmaceutical Strategy for Europe [35]. Importantly, environmental contamination from human and animal waste, pharmaceutical production and use of antimicrobial crops will need further consideration and concerted global action [36, 37]. In order to influence policy and action, the broadening of systems linking AMR and prescribing is required, building on existing initiatives such as the World Health Organization’s (WHO) Global AMR Surveillance System (GLASS) and the repository for surveillance of animal data, resistancebank.org [33, 38]. In this respect, environmental assessment and monitoring are additional tools aiding in closing the existing knowledge gap [39, 40].

### Priorities

Significantly, access to clean water, sanitation and high-quality healthcare are acknowledged as crucial public health interventions for prioritization [41]. Raising awareness of the AMR challenges, policy incentives and adequate regulation all play a role in combatting resistance. As part of the research agenda, the development of rapid diagnostics, vaccines and new antimicrobial agents, including alternative therapeutics (such as bacteriophages, monoclonal antibodies, virulence factor modulating products) need to be incentivized. In this respect, Non-Profit Partnership and Foundation initiatives play an important role as “push” incentives: e.g. Combating Antibiotic-Resistant Bacteria Biopharmaceutical Accelerator (CARB-X) (https://carb-x.org/), and the Replenishing and Enabling the Pipeline for Anti-Infective Resistance (REPAIR) Impact Fund (https://www.repair-impact-fund.com/) address discovery and early phases of development, whilst the Global Antibiotic Research and Development Partnership (GARDP) aims to take development through the later stages (https://gardp.org/) [42]. In order to address inadequate economic incentives for pharmaceutical product developers, the need for alternative payment models, decoupling profits from antimicrobials sales volume is well appreciated (“pull” incentive). The United Kingdom is currently trialing a pioneering scheme with a subscription model, using a health technology assessment process that establishes a “base value” to be paid to pharmaceutical companies for innovative antimicrobials (with key focus on serious infections, such as HAP). This scheme is the first of its kind and further confirmation of its intended effect is awaited [43, 44].

Notwithstanding the above mentioned initiative, it is generally advisable that information needed by health technology assessment bodies to assess the added value of new antibiotics,—in particular to tackle multi-drug resistant infections—, is collected during the development phase, to facilitate and potentially accelerate access to new antimicrobials. In the EU, the European Medicines Agency introduced some flexibility with the possibility for limited clinical development programmes for medicines that will benefit patients with multi-drug resistant infections [45, 46]. Alignment of data requested by regulators across regions, has been a further endeavour intended to help stimulate development of new antibiotics to fight AMR [47]. Additionally, the European medicines agencies network strategy to 2025 intends to build further on the existing initiatives and to strengthen the measures in combatting AMR [48]. The network identified six main goals related to AMR and other emerging health threats, to be achieved within the reference period (Table 1).

**Table 1:** EMA/HMA: strategic goals for AMR and other emerging health threats. *Source*: European Medicines Agencies Network (EMA/HMA), 2020

From: European medicines agencies network strategy to 2025: Protecting public health at a time of rapid change (Ref. [48])
Provide high quality information on antimicrobial consumption and surveillance data on antimicrobial resistance in animals and humans in support of policy development
Contribute to responsible use of antibacterial agents and effective regulatory antimicrobial stewardship in human and veterinary sectors by putting in place strategies to improve their use by patients, healthcare professionals and national authorities
Ensure regulatory tools are available that guarantee therapeutic options (with a focus on veterinary medicines) while minimising impact of antimicrobial resistance on public health and the environment
Define pull incentives for new and old antibacterial agents, including investigating support for new business models and not-for-profit development
Foster dialogue with developers of new antibacterial agents and alternatives to traditional antimicrobials, to streamline their development and provide adequate guidance in both human and veterinary medicine
Improve regulatory preparedness for emerging health threats

*Inter alia*, the importance of continued collaboration with other actors and stakeholders is deemed vital, e.g. in reviewing the pipeline of investigational antibacterial agents and essential “old antibiotics”, and further engagement in international forums is stressed to ensure harmonisation. It is also recognised that AMR national action plans within the EU remain varied and largely uncoordinated so far and hence, one of the challenges for the network will be to leverage national efforts, to foster their alignment and to further refine a targeted common European approach on AMR. In addition, it is acknowledged that beyond AMR, the emergence and re-emergence of infectious diseases requires a global collaboration to develop effective and timely responses [46, 48]. For these, a One Health approach will often best address such threats, with the experiences in tackling AMR providing valuable reference points.

## Conclusions

Although some achievements can be counted, there is still a long battle to go to keep pace with newly emerging resistance to antibacterials and antifungals. From the global health perspective, AMR is a problem which mandates an integrated multisectoral approach with appropriate financial support to find a satisfactory solution. COVID-19 is part and parcel of a same phenomenon with the sudden emergence of SARS-CoV-2 to be viewed through a likewise prism as AMR: a holistic, ecological approach is not amiss to avert future biological health threats to mankind [49].

## Data Availability

Not applicable.

## Abbreviations

AMR: Antimicrobial resistance
CARB-X: Combating antibiotic-resistant bacteria biopharmaceutical accelerator
COVID-19: Coronavirus disease 2019
CREB: Carbapenem-resistant *Enterobacteriaceae*
EMA/HMA: European medicines agencies network
ESVAC: European Surveillance of Veterinary Antimicrobial Consumption
EU: European Union
GARDP: Global Antibiotic Research and Development Partnership
GLASS: Global AMR Surveillance System
HAP: Hospital acquired pneumonia
ICU: Intensive care unit
LMIC: Lower-and-middle-income country
OTC: Over the counter
REPAIR: Replenishing and Enabling the Pipeline for Anti-Infective Resistance
SARS-CoV-2: Severe Acute Respiratory Syndrome Coronavirus 2
VAP: Ventilator acquired pneumonia
WHO: World Health Organization
